# Cost-effectiveness of sintilimab plus chemotherapy versus chemotherapy alone as first-line treatment of locally advanced or metastatic oesophageal squamous cell carcinoma

**DOI:** 10.3389/fimmu.2023.1092385

**Published:** 2023-01-23

**Authors:** Lulu Liu, Lei Wang, Li Chen, Yiling Ding, Qilin Zhang, Yamin Shu

**Affiliations:** ^1^ Department of Pharmacy, Tongji Hospital, Tongji Medical College, Huazhong University of Science and Technology, Wuhan, China; ^2^ School of Pharmaceutical Science and Technology, Tianjin University, Tianjin, China; ^3^ Department of Pharmacy and Evidence-Based Pharmacy Center, West China Second University Hospital, Sichuan University, Chengdu, China; ^4^ Graduate School of Pharmaceutical Sciences, University of Tokyo, Tokyo, Japan; ^5^ Department of Pharmacy, Union Hospital, Tongji Medical College, Huazhong University of Science and Technology, Wuhan, China

**Keywords:** cost-effectiveness, immunotherapy, oesophageal squamous cell carcinoma, quality-adjusted life years, sintilimab

## Abstract

**Background:**

Sintilimab plus chemotherapy significantly prolongs overall survival (OS) for patients with advanced or metastatic oesophageal squamous cell carcinoma (OSCC). However, the cost-effectiveness of this high-priced therapy is currently unknown. We evaluated the cost-effectiveness of sintilimab plus chemotherapy vs chemotherapy alone as fist-line therapy in patients with advanced or metastatic OSCC from the perspective of Chinese healthcare system.

**Methods:**

A partitioned survival model consisting of 3 discrete health states was constructed to assess the cost and effectiveness of sintilimab plus chemotherapy vs chemotherapy as first-line treatment of OSCC. Key clinical data in the model came from the ORIENT-15 trial. Costs and utilities were collected from published sources. Life-years, quality-adjusted life-years (QALYs), incremental cost-effectiveness ratio (ICER), incremental net health benefits (INHB), and incremental net monetary benefits (INMB) were calculated for the two treatment strategies. One-way and probabilistic sensitivity analyses were conducted to account for uncertainty and model stability. Additional subgroup and scenario analyses were performed.

**Results:**

Treatment with sintilimab plus chemotherapy provided an additional 0.37 QALYs and an incremental cost of $8,046.58 compared with chemotherapy, which resulted in an ICER of $21,782.24 per QALY gained. One-way sensitivity analysis revealed that the model was most sensitive to utility of progression-free survival (PFS) and the cost of sintilimab. The probabilistic sensitivity analysis indicated that the probability of sintilimab plus chemotherapy being cost-effective was 0.01%, 76.80% and 98.60% at the threshold of 1, 2 or 3 times GDP per capita per QALY, respectively. Subgroup analysis found that all subgroups other than PD-L1 expression combined positive scores < 1 subgroup favored sintilimab plus chemotherapy treatment due to its association with positive INHBs by varying the hazard ratios for OS and PFS. The scenario analyses showed altering the time horizon of the model or fitting survival curves separately did not reverse results of the model.

**Conclusion:**

Sintilimab plus chemotherapy was associated with improved QALYs and an additional cost but was estimated to be cost-effective compared with chemotherapy alone as a first-line treatment for patients with advanced or metastatic OSCC at the commonly adopted willingness-to-pay threshold of 3 times GDP per capita per QALY in China.

## Introduction

Oesophageal squamous cell carcinoma (OSCC) is the predominant histological subtype of oesophageal cancer in Asian populations (approximately 90%), whereas oesophageal adenocarcinoma is more common in North America and western European countries (1, 2). According to the survey statistics of the International Agency for Research on Cancer in 2020, there were 324,422 new cases of oesophageal cancer and 301,135 deaths in China, which accounted for 53.70% and 55.35% of the global incidence and mortality of esophageal cancer, respectively (3).

Currently, platinum doublet chemotherapy is commonly the most recommended first-line therapy for patients with unresectable advanced or metastatic OSCC (4, 5). However, the overall survival (OS) remains limited with a median of less than 12 months, and the 5-year survival rate is only 8% or less for patients diagnosed with advanced stages (6). Accumulating studies have shown that immune checkpoint inhibitors (ICIs) combined with chemotherapy demonstrated superior anti-tumor effects compared with chemotherapy alone (7–9). Sintilimab is a fully recombinant human IgG4 anti-PD-1 monoclonal antibody that inhibits binding of the PD-1 receptor to its ligands (PD-L1 and PD-L2), thereby restoring T-cell immune activity and reversing the immune evasion of cancer (10). Compared with pembrolizumab or nivolumab, sintilimab has a different binding site and potentially greater affinity against PD-1 (11).

The multicentre, double blind, randomized phase 3 clinical trial ORIENT-15 conducted at 79 sites in five countries, reported the efficacy and safety of sintilimab plus chemotherapy compared with placebo plus chemotherapy for the first-line treatment of locally advanced or metastatic OSCC (12). Results indicated that sintilimab plus chemotherapy significantly prolonged the progression-free survival (PFS) (7.2 vs 5.7 months; hazard ratio [HR], 0.56; 95% CI, 0.46-0.68; *P*<0.001) and OS (median 16.7 vs 12.5 months; HR, 0.63; 95% CI, 0.51-0.78; *P*<0.001) compared with placebo plus chemotherapy. Treatment-related adverse events (AEs) of grade 3 or higher were comparable between the 2 groups (60% vs 55%). Overall, the current data support sintilimab plus chemotherapy as an attractive first-line treatment option for patients with advanced OSCC. Excitedly, the guideline of Chinese Society of Clinical Oncology (CSCO) for the diagnosis and treatment of esophageal cancer (2022 edition) published on April, 2022, has recommended sintilimab in combination with paclitaxel and cisplatin as a first-line treatment option for advanced or metastatic OSCC (13).

Notwithstanding the excellent clinical benefits of sintilimab in first-line OSCC therapy, its cost-effectiveness has not, to our knowledge, been evaluated in China and other countries. The economic evaluation of sintilimab is critical and helpful for clinicians and decision-makers to optimally allocate limited health resources. The objective of our study is to estimate the cost-effectiveness of sintilimab plus chemotherapy vs chemotherapy alone as first-line treatment for patients with advanced or metastatic OSCC based on the ORIENT-15 trial data from the perspective of Chinese healthcare system.

## Methods

### Analytic overview

**Figure 1:**
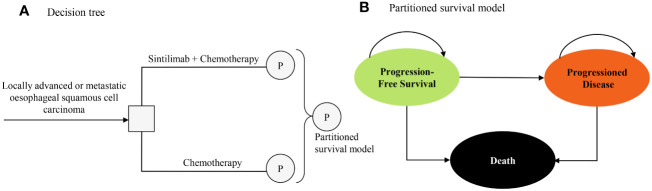
Model structure of a decision tree combining the partitioned survival model with the 3 health states. **(A)** Decision tree. **(B)** Partitioned survival model.

The target population for this analysis was patients who had locally advanced or metastatic oesophageal squamous cell carcinoma and did not receive previous systemic treatment, consistent with the patient characteristics in the ORIENT-15 trial (12). A partitioned survival model with 3 health states was developed to conduct the economic analyses from a Chinese healthcare system perspective, and the perspective is consistent with the objective of this study (to estimate the cost-effectiveness of sintilimab plus chemotherapy and help decision-makers in healthcare system to optimally allocate limited health resources) (14). The three mutually exclusive health states were progression-free survival (PFS), progressed disease (PD), and death (**Figure 1**). In the model, the proportion of patients in each health state at each cycle t was estimated from OS and PFS curves of the ORIENT-15 trial (12). The proportion of patients alive at each cycle (1-week cycle) was estimated by the area under the OS curve, and the proportion alive with PFS was estimated by the area under the PFS curve (15). The proportion of PD was estimated by the difference between the OS and PFS curves (15). The time horizon was 11 years given that more than 99% of the patients died at this time point, and 5 to 20 years were included in the scenario analyses.

This study followed the reporting guideline of Consolidated Health Economic Evaluation Reporting Standards (CHEERS) (**Supplementary Table 1**) (16). This evaluation was based on a literature review of publicly available data and on modeling techniques, which did not require institutional review board review or exemption by the ethics committee.

### Clinical data inputs

To construct the partitioned survival model, graphic data from the ORIENT-15 trial were extracted by GetData Graph Digitizer, version 2.18, and time-to-event data were obtained as described in the study by Guyot et al. (17). The original and reconstructed Kaplan–Meier curves were shown in **Supplementary Figure 1**, and summary measures regarding median duration and survival rate in original publication and reconstructed data were shown in **Supplementary Table 2**. Because time horizon in this economic study was 11 years (99% of the patients died) beyond the follow-up period in ORIENT-15 trial, the OS and PFS curves of chemotherapy alone were fitted and extrapolated using the following parametric survival functions: Weibull, log-normal, log-logistic, exponential, generalized gamma, Gompertz, and Royston-Parmar spline models and parametric mixture and non-mixture cure models (18–20). The eligible survival function was chosen based on the visual inspection, and lowest value of the Akaike information criterion (AIC) and Bayesian information criterion (BIC) (18). The proportional hazards (PH) modelling method was used to estimate the parametric PFS and OS curves for the sintilimab plus chemotherapy group based on the hazard ratios (HRs) of sintilimab plus chemotherapy vs chemotherapy alone reported in ORIENT-15 trial (12). This could avoid the bias resulted from inconsistency of parametric survival distribution between two groups (18).

The final survival functions of the sintilimab plus chemotherapy vs chemotherapy alone were shown in **Table 1** and the goodness-of-fit results were shown in **Supplementary Table 3**. The proportions of patients with PFS and OS were calculated by using the selected survival distribution. The validation plot, survival distribution, were shown in **Supplementary Figure 2**, **3**. The key clinical inputs were listed in **Table 1**.

**Table 1:** Key Model Inputs.

Parameter	Base case (Range)	Distribution	Source
Clinical input			
Survival model for chemotherapy			
Log-logistic model for PFS	shape = 2.16, scale = 5.73	Fixed	(12)
Gamma model for OS	shape = 1.87, rate = 0.12	Fixed	(12)
Survival model for sintilimab plus chemotherapy			
Royston-Parmar model for PFS^a^	gamma0 = -4.84, gamma1 = 2.42, gamma2 = -0.44, gamma3 = 0.74	Fixed	(12)
Royston-Parmar model for OS^a^	gamma0 = -5.08, gamma1 = 1.34, gamma3 = -1.26, gamma3 = 1.72	Fixed	(12)
HR for PFS associated with sintilimab plus chemotherapy vs placebo plus chemotherapy	0.56 (0.46 to 0.68)	Log-normal	(12)
HR for OS associated with sintilimab plus chemotherapy vs placebo plus chemotherapy	0.63 (0.51 to 0.78)	Log-normal	(12)
Costs input ($)			
Sintilimab per 100 mg^b^	167.44 (125.58 to 209.30)	Gamma	(21)
Cisplatin per 100 mg^c^	43.19 (32.39 to 53.99)	Gamma	(21)
Paclitaxel per 100 mg^c^	120.93 (90.70 to 151.16)	Gamma	(21)
5-fluorouracil per 100 mg^c^	8.91 (6.68 to 11.14)	Gamma	(21)
Proportion of receiving paclitaxel-based chemotherapy	6.53% (4.90%-8.16%)	Beta	(12)
Cost of intravenous drug administration per unit^d^	134.93 (101.20 to 168.66)	Gamma	(22)
Cost of laboratory tests and scans per cycle	83.21 (62.41 to 104.01)	Gamma	(23)
Cost of subsequent best supportive care per cycle	39.03 (29.27 to 48.79)	Gamma	(23)
Cost of routine follow-up per cycle	17.16 (12.87 to 21.45)	Gamma	(23)
Cost of terminal care in end-of-life	1,460.30 (1,09.23-1,825.38)	Gamma	(23)
Utility value			
Utility in PFS	0.74 (0.56 to 0.93)	Beta	(22)
Utility in PD	0.58 (0.44 to 0.73)	Beta	(22)
AEs disutility			
Grade 1 and 2	0.09 (0.07 to 0.11)		(24)
Grade 3 and higher	0.20 (0.15 to 0.25)		(24)
Others			
Body surface area (m^2^)	1.72 (1.38 to 2.06)	Normal	(23)
Discount rate (%)	5% (0% to 8%)	Beta	(25)

AE, adverse event; HR, hazard ratio; OS, overall survival; PD, progressed disease; PFS, progression-free survival.

a Survival function for sintilimab plus chemotherapy (Royston-Parmar distribution) was only used in scenario analysis.

b Treatment with sintilimab continued until disease progression, or 2 years of follow-up.

c Treatment with chemotherapy continued until a maximum of 18 weeks.

d Including the cost of preventive medication per administered intravenously, infusion fee per administered intravenously and hospitalization fee per administered intravenously.

### Cost and utility inputs

Only direct medical costs within the healthcare system were included in this study (**Table 1**), including costs of acquiring and administrating drugs, costs of subsequent treatment after disease progressed, costs for routine follow-up, laboratory tests and scans, costs for end-of-life care and costs for the management of AEs. All costs were reported in 2021 US dollars with the exchange rate in 2021: $1 = ¥6.45, and were adjusted to 2021 value using the consumer price index (26).

According to the ORIENT-15 trial report, sintilimab was given intravenously at a dose of 200 mg every 3 weeks, and it was continued until progressive disease, lost to follow-up, death, or a maximum of 2 years, whichever occurred first (12). And 6.53% patients would receive cisplatin (75 mg/m^2^ every 3 weeks) plus paclitaxel (87.5 mg/m^2^ on the first and second weeks; 175 mg/m^2^ every 3 weeks starting from the fourth week) as chemotherapy regimen, and other patients (93.47%) would receive cisplatin plus 5-fluorouracil (4000 mg/m^2^ every 3 weeks) as chemotherapy regimen (12). Chemotherapy was given within a maximum of 18 weeks (12). To calculate the dosage of paclitaxel, we assumed that the body surface area was 1.72 m^2^ for a typical patient in China (23). The prices of sintilimab, cisplatin, paclitaxel, and 5-fluorouracil were collected from Chinese bid-winning price (21).

After disease progression, 41.0% patients in the sintilimab plus chemotherapy group and 54% in the chemotherapy alone group received subsequent systemic medication therapy, and the proportion and costs for each subsequent therapy were shown in **Supplementary Table 4**, which were estimated from ORIENT-15 trial and Chinese bid-winning price (21). The costs of laboratory tests and scans ($134.93 per cycle), costs of subsequent best supportive care ($39.03 per cycle), cost of routine follow-up ($17.16 per cycle) and cost of terminal care in end-of-life ($1,460.30 per patient) were collected from an economic evaluation among patients with advanced OSCC. The analysis included the costs associated with management of grade 3 or higher AEs (probability ≥ 5%), which were extracted from the literature (**Supplementary Table 5**).

Each health state was assigned a health utility preference on a scale of 0 (death) to 1 (perfect health). The PFS and PD states associated with OSCC were 0.74 and 0.58 respectively, which were derived from a cost-effectiveness analysis considering patients with OSCC (22). The disutility values due to grade 1 or 2 and grade 3 or higher AEs were included in this analysis (24). All AEs were assumed to be incurred during the first cycle.

### Base-case analysis

The incremental cost-effectiveness ratio (ICER) was calculated as the incremental cost per additional quality adjusted life-year (QALY) gained between the sintilimab plus chemotherapy group and the chemotherapy alone group. When the ICER was lower than the prespecified willingness-to-pay threshold (WTP, 3 times GDP per capita per QALY gained, $37,663.26/QALY), cost-effectiveness was assumed according to the recommendation of Guidelines for Evaluation of Chinese Pharmacoeconomics (25). Costs and QALYs were discounted at an annual rate of 5% (25).

The incremental net health benefit (INHB) and incremental net monetary benefits (INMB) were also estimated based on the following formulas: INHB (λ) = (μ*_E_*_1_ − μ*_E_*_0_) − (μ*_C_*_1_ − μ*_C_*_0_)/λ = Δ*E* − Δ*C*/λ; INMB (λ) = (μ*_E_*_1_ − μ*_E_*_0_) × λ − (μ*_C_*_1_ − μ*_C_*_0_) = Δ*E* × λ − Δ*C*, where μ*_Ci_* and μ*_Ei_* were the costs and effectiveness of sintilimab plus chemotherapy (*i* = 1) or chemotherapy (*i* = 0), respectively, and λ was the WTP threshold (27, 28).

### Sensitivity, subgroup, and scenario analyses

To evaluate the robustness of the base-case results, we conducted one-way and probabilistic sensitivity analyses. One-way sensitivity analyses were conducted for all parameters, and the estimated range of each parameter was based on either the reported or estimated 95% CIs in the referenced studies or determined by assuming a 25% change from the base-case value (**Table 1**). In the probabilistic sensitivity analyses, a Monte Carlo simulation with 1000 iterations was generated by simultaneously sampling the key model parameters from the prespecified distributions. A gamma distribution was selected for the cost parameters, a log-normal distribution for the HRs, and a beta distribution for proportion, and health utility parameters. Based on the data from 1000 iterations, a cost-effectiveness acceptability curve was created to represent the probability that sintilimab plus chemotherapy would be considered cost-effective at various WTP thresholds for health gains (QALYs). To investigate the uncertainty of economic outcomes caused by the subpopulations, exploratory subgroup analyses were performed for the prespecified subgroups that were reported in the ORIENT-15 trial by varying the HRs for OS and PFS. Lastly, additional scenario analyses were conducted: 1) time horizon was 5 or 20 years; 2) four survival curves (OS and PFS curves for sintilimab plus chemotherapy vs chemotherapy alone) were fitted separately to estimate parametric survival curve. Survival curves were fitted with flexsurv packages in R, version 4.1.1, 2021, and the partitioned survival model was developed in Microsoft Excel 2019.

## Results

### Base-case analysis

In comparison with chemotherapy alone, sintilimab plus chemotherapy provided an additional 0.37 QALYs and 0.50 overall life-years, with an incremental cost of $8,046.58, which was associated with an ICER of $21,782.24/QALY. The INHB was +0.16 QALYs, and the INMB was +$5,866.61 at a WTP threshold of 3 times GDP per capita per QALY (**Table 2**).

**Table 2:** Summary of Cost and Outcome Results in the Base-Case Analysis.

Variables	Sintilimab plus chemotherapy	Chemotherapy alone
Cost, $		
First-line drug	8,837.87	3,619.66
Overall	24,508.98	16,462.40
Effectiveness		
Life-years		
Progression-free	1.11	0.62
Overall	1.65	1.15
QALYs	1.12	0.75
**Incremental cost per QALY**	21,782.24	NA
INHB, QALY^a^		
λ=1 times GDP	-0.27	NA
λ=2 times GDP	0.05	NA
λ=3 times GDP	0.16	NA
INMB, $^a^		
λ=1 times GDP	-3,408.85	NA
λ=2 times GDP	1,228.88	NA
λ=3 times GDP	5,866.61	NA

INHB, incremental net health benefit; INMB, incremental net monetary benefit; NA, not applicable; QALY, quality-adjusted life-years.

a Compared with chemotherapy alone.

### Sensitivity analysis

**Figure 2:**
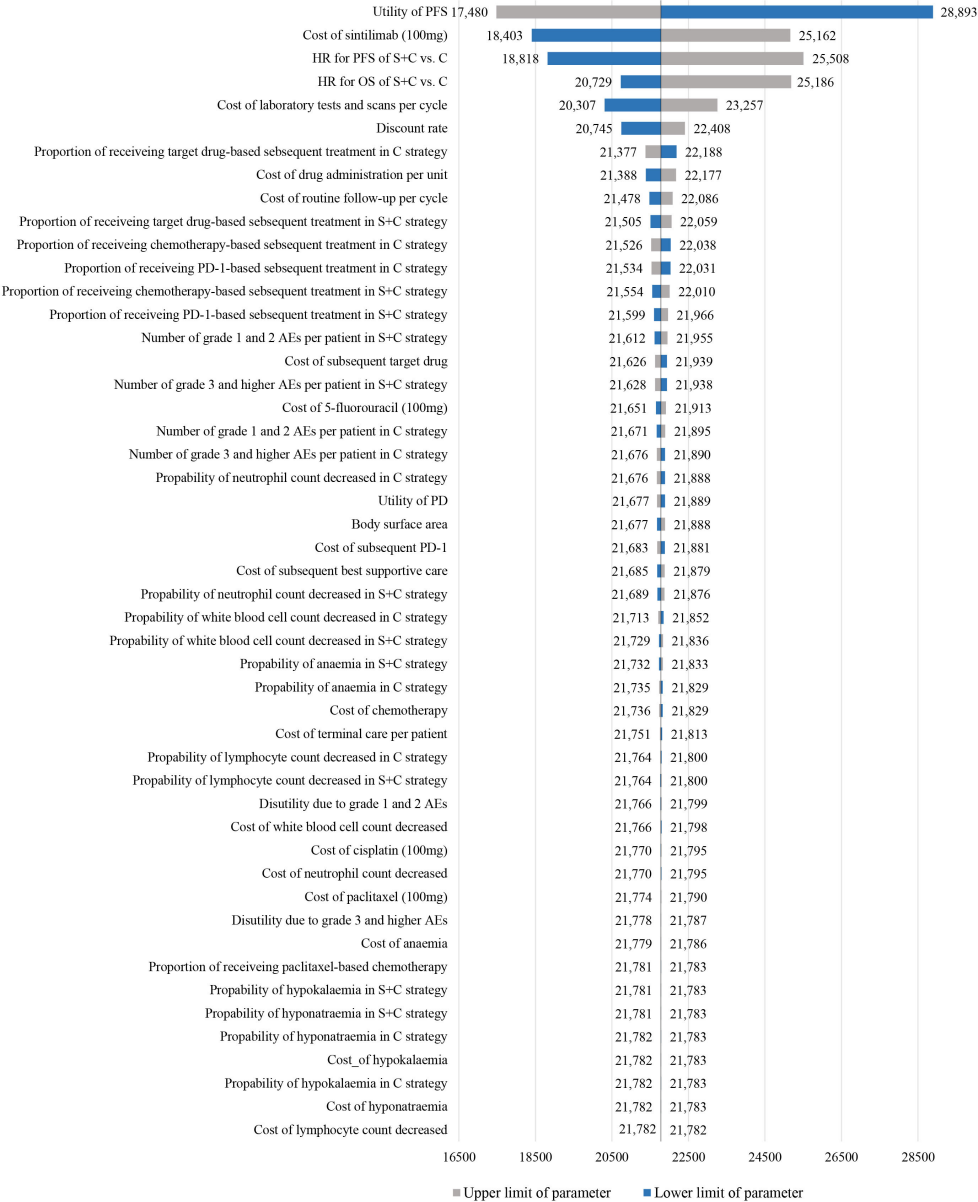
Tornado diagram of one-way sensitivity analyses of sintilimab plus chemotherapy vs chemotherapy alone in order of magnitude of the association.

One-way sensitivity analyses showed that the model was particularly sensitive to utility of PFS and the cost of sintilimb (**Figure 2**). When the lower boundary of the utility (0.56) for PFS was adopted, the ICER of sintilimab plus chemotherapy vs chemotherapy alone was $28,892.78/QALY, and when the upper boundary (0.93) was adopted, the ICER was $17,480.31/QALY. When the cost of sintilimab was discounted by 25%, the ICER was $18,402.56/QALY. The remaining parameters, such as the HR (for PFS and OS), cost and utility related to AEs, had only moderate or low associations with the ICER. All parameters were not associated with ICERs exceeding the threshold.

**Figure 3:**
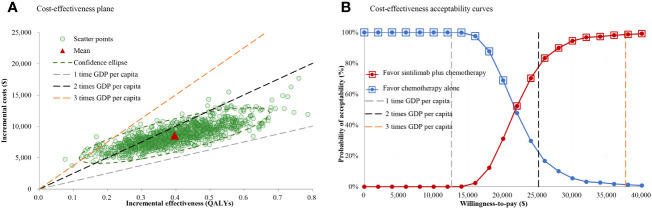
Cost-effectiveness plane and acceptability curves for sintilimab plus chemotherapy vs chemotherapy alone. **(A)** Cost-effectiveness plane. **(B)** Cost-effectiveness acceptability curves.

Compared with chemotherapy alone, the probabilistic sensitivity analysis showed that sintilimab plus chemotherapy added a mean of 0.40 QALYs with an additional mean cost of $8.560.06, which resulted in a mean ICER of $21,621.89/QALY (**Figure 3A**). The cost-effectiveness acceptability curve showed that the probability of sintilimab plus chemotherapy being cost-effective was 0.01%, 76.80% and 98.60% at 1, 2- or 3-times GDP per capita per QALY threshold, respectively (**Figure 3B**).

### Subgroup analyses

**Figure 4:**
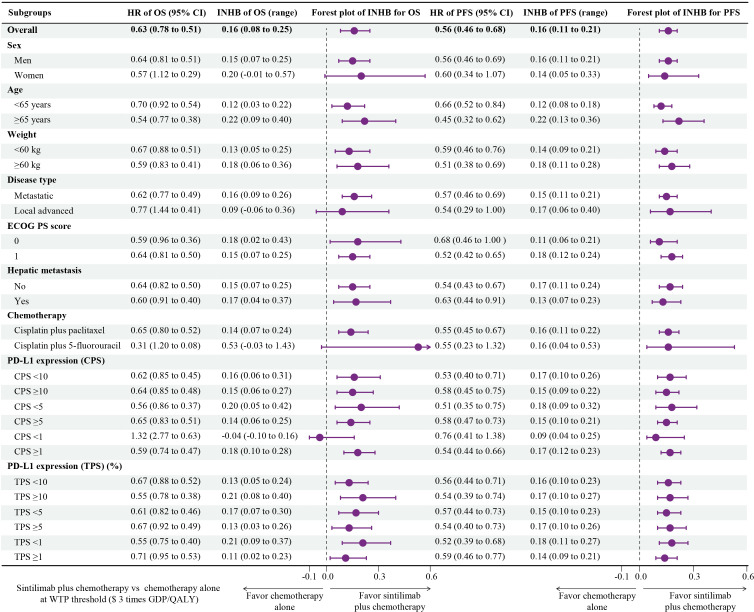
Subgroup Analysis Results of Incremental Net Health Benefits (INHBs) Obtained by Varying the Hazard Ratios (HRs) for Overall Survival and Progression-Free Survival.

The subgroup analyses, which were conducted by varying the HRs for OS and PFS, revealed that sintilimab plus chemotherapy was associated with primarily positive INHBs in the most subgroups at the threshold of 3-times GDP per capita per QALY (**Figure 4**). Only among patients with CPS<1, the point estimate of INHBs was negative, and in fewer subgroups (women, local advanced, cisplatin plus 5-fluorouracil, CPS<1), the lower bound of INHBs by varying the HRs for OS were negative.

### Scenario analyses

When the time horizon was 5 or 20 years, the overall cost of sintilimab plus chemotherapy was $23,671.40 or $24,909.54, resulting in an ICER of sintilimab plus chemotherapy vs chemotherapy alone of $ 22,919.51 or $21,680.02 per QALY. When four survival curves (OS and PFS curves for sintilimab plus chemotherapy vs chemotherapy alone) were fitted separately, the overall cost of sintilimab plus chemotherapy was $26,784.74, resulting in an ICER of $18,329.16/QALY (**Table 3**).

**Table 3:** Results of Scenario Analyses.

Variables	Costs	QALYs	ICERs
Time horizon (5 years)			
Sintilimab plus chemotherapy	23,671.40	1.06	22,919.51
Chemotherapy alone	16,347.39	0.74	NA
Time horizon (20 years)			
Sintilimab plus chemotherapy	24,909.54	1.14	21,680.02
Chemotherapy alone	16,489.40	0.75	NA
Survival curves were fitted separately			
Sintilimab plus chemotherapy	26,784.74	1.31	18,329.16
Chemotherapy alone	16,462.40	0.75	NA

ICERs, Incremental Cost-Effective Ratio; NA, not applicable; QALY, quality-adjusted life-years.

## Discussion

At present, ICIs alone or in combination with other regimens has becoming a prominent option for the treatment of advanced oesophageal cancer. However, there are few studies on the pharmacoeconomic evaluation of immunotherapies for OSCC. Based on our model, sintilimab plus chemotherapy cost $21,782.24 per additional QALY gained compared with chemotherapy alone, suggesting sintilimab plus chemotherapy would be considered cost-effective when assuming a WTP threshold at 2- or 3-times GDP per capita per QALY. Results were generally robust, as evidenced by the results of one-way sensitivity analysis and probabilistic sensitivity analysis. At a WTP threshold of 3 times GDP per capita per QALY, all subgroups other than CPS<1 subgroup favored sintilimab plus chemotherapy treatment due to its association with positive INHBs by varying the HRs for OS and PFS compared with chemotherapy alone. Economic evaluations of the 26 subgroups may be useful in tailoring treatment decisions by physicians, patients and policy makers.

Interestingly, different PD-1 inhibitors showed completely distinct cost-effectiveness in the first-line treatment of OSCC. Contrary to our previous study, camrelizumab plus chemotherapy was not a cost-effective option as the first-line treatment for advanced or metastatic OSCC because the ICER of camrelizumab plus chemotherapy versus chemotherapy was $46,671.10 per QALY gained, which was higher than the WTP threshold of China (23). Another two recent studies reported by Wu et al. (29) and Zheng et al. (30) investigated the cost-effectiveness of pembrolizumab plus chemotherapy vs placebo plus chemotherapy in the first-line therapy of oesophageal cancer patients in China, and also obtained results contrary to the current study with ICERs of $115,391.84/QALY and $41,805.12/QALY, respectively, exceeding the threshold of WTP. These opposite results to ours maybe owing to different trial data and survival curve simulation techniques and different costs of PD-1 inhibitors, leading to various incremental costs and QALYs. In these settings, clinicians and decision-makers can leverage knowledge of economics to advance policy discussions about health care costs and to take action on accessibility, affordability, and value for use of these novel immunotherapies. Although there is no cost-effectiveness analysis study of sintilimab in the treatment of OSCC, some studies have reported that sintilimab plus chemotherapy is cost-effective in the first-line therapy of locally advanced or metastatic non-small cell lung cancer (31, 32). Moreover, the combination of sintilimab plus bevacizumab is also likely to be a cost-effective option compared with sorafenib as the first-line treatment of patients with unresectable hepatocellular carcinoma in China (33–35).

Results of the one-way sensitivity analysis confirmed that the model was most sensitive to the utility of PFS and cost of sintilimab. When the cost of sintilimab was reduced, sintilimab plus chemotherapy treatment would become more favorable because its ICER was lower than 1 times GDP per capita per QALY. After that, HR for PFS and OS had the greatest effect on the model results, demonstrating sintilimab plus chemotherapy was more cost-effective for patients with a favorable prognosis, such as patients with age ≥ 65 years and weight ≥ 60 kg. Notably, as these parameters varied within the specified range, ICERs dramatically increased or decreased, which induced ICERs still below the WTP threshold of 3 times GDP per capita per QALY, making sintilimab plus chemotherapy consistently cost-effective, which was further supported by the cost-effectiveness acceptability curve that the probability of sintilimab plus chemotherapy being cost-effective was 76.80% and 98.60% at 2 or 3 times GDP per capita per QALY threshold, respectively. Furthermore, the scenario analyses also affirmed the stability of the model results. Although altering the time horizon of the model or fitting survival curves separately, the ICERs remained below 2 times GDP per capita per QALY ($25,108.84/QALY).

### Strengths and limitations

The strengths of this study are worth highlighting. To the best of our knowledge, this study is the first to evaluate the economic outcomes of sintilimab plus chemotherapy in the first-line treatment for advanced or metastatic OSCC by integrating the latest evidence through a pharmacoeconomic modeling technology. Besides, the partitioned survival model is employed in this economic analysis for the advantages that survival curve can be directly used to obtain the proportion of patients with different health states, and complex risk functions can be directly reconstructed by extrapolation method, so the calculation is relatively simple. Furthermore, there is no need to make additional assumptions on the model to calculate the probability of transition between states, so it is more suitable for the actual survival of patients.

There are also several limitations in our study. First, we only evaluated the cost-effectiveness of sintilimab plus chemotherapy vs chemotherapy alone for patients with advanced OSCC. In fact, the cost-effectiveness of sintilimab vs similar drugs (other ICIs) should also be assessed. However, due to the lack of robust head-to-head trial data, we could not compare the economics of other immunotherapies with that of sintilimab directly, such as pembrolizumab, camrelizumab, and nivolumab, which had also shown favorable health benefits for patients with advanced OSCC. Therefore, future research will perform network meta-analyses to obtain HRs for OS and PFS between different ICIs regimens, subsequently achieving cost-effectiveness comparisons. Second, though ORIENT-15 is a large and well-designed trial, any biases such as rigorous eligible patients, high medication adherence and racial difference, can affect its validity and generalizability. Thus, there’s a difference in generalizing our cost-effectiveness analysis results outside the Chinese setting. Third, clinical benefits beyond the observation time of the ORIENT-15 trial were assumed through fitting of parametric distributions to the reported Kaplan-Meier PFS and OS data, which may lead to uncertainty in the model outcomes, although flexible parametric models were applied and the modeled and observed data were validated. However, we have conducted a series of sensitivity, subgroup and scenario analyses to evaluate the uncertainty, and they showed the model results were robust. Fourth, the costs of grade 1 and 2 AEs were excluded, which might result in the overestimation of the economic results associated with sintilimab plus chemotherapy. Lastly, the disutility value for AEs extracted from a study outside Chinese setting, may also cause some bias. These limitations may not be major factors, as showed in the one-way sensitivity analysis indicating that the influence of the costs/disutility values of AEs were minor.

## Conclusion

This economic evaluation showed that sintilimab plus chemotherapy was associated with improved QALYs and an additional cost but was estimated to be cost-effective compared with chemotherapy as a first-line treatment for patients with advanced or metastatic OSCC at the commonly adopted WTP threshold of 3 times GDP per capita per QALY in China. The findings may help clinicians and oncologists determine the preferred economic treatment strategy of advanced OSCC.

## Data availability statement

The original contributions presented in the study are included in the article/**Supplementary Material**. Further inquiries can be directed to the corresponding authors.

## Author contributions

YS and QZ conceived and administered the study. LL, QZ, and YS conducted the analysis, drafted the manuscript and interpreted the results. LW and YD substantively revised the manuscript. All authors contributed to the article and approved the submitted version.
