# Supplementary material for: Cost-effectiveness of sintilimab plus chemotherapy versus chemotherapy alone as first-line treatment of locally advanced or metastatic oesophageal squamous cell carcinoma
Source: Front Immunol. 2023 Jan 23;14:1092385. doi: 10.3389/fimmu.2023.1092385 (PMC9899904; doi:10.3389/fimmu.2023.1092385)
Supplement: Supplementary file 1 [file DataSheet_1.docx]

**Supplementary Materials**

**Supplementary Figure 1. Visual Inspection of Original and Reconstructed Kaplan–Meier curves.**

**Supplementary Figure 2. The Parametric PFS Curves of Sintilimab plus Chemotherapy vs Chemotherapy in the ORIENT-15 Trial.**

**Supplementary Figure 3. The Parametric OS Curves of Sintilimab plus Chemotherapy vs Chemotherapy in the ORIENT-15 Trial.**

**Supplementary Table 1. CHEERS 2022 Checklist.**

**Supplementary Table 2. Comparison of summary measures between original publication and reconstructed individual data**

**Supplementary Table 3. Estimated Parameters and Goodness of Fit from Each Survival Model.**

**Supplementary Table 4. Probability and Costs Related to Subsequent Therapy.**

**Supplementary Table 5. Probability and Costs Related to Adverse Events.**

**Supplementary Figure 1. Visual Inspection of Original and Reconstructed Kaplan–Meier curves. (A) Original overall survival curves from the ORIENT-15 trial. (B) Reconstructed overall survival curves. (C) Original progression free survival curves from the ORIENT-15 trial. (D) Reconstructed progression free survival curves.**

According to the results of visual inspection, we found the reconstructed KM curves and number at risk were in close proximity to original publication.


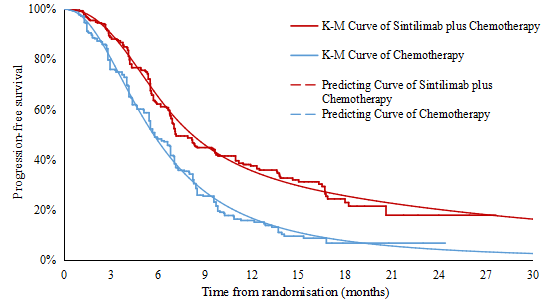


**Supplementary Figure 2. The Parametric PFS Curves of Sintilimab plus Chemotherapy vs Chemotherapy in the ORIENT-15 Trial.**


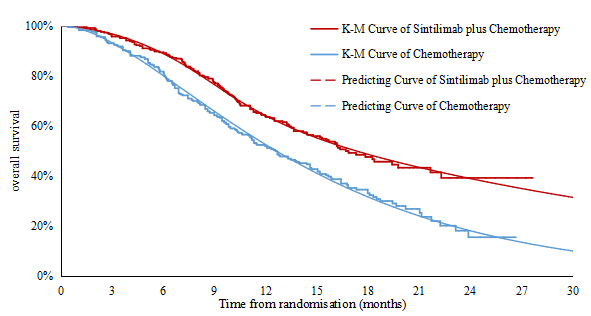


**Supplementary Figure 3. The Parametric OS Curves of Sintilimab plus Chemotherapy vs Chemotherapy in the ORIENT-15 Trial.**

**Supplementary Table 1. CHEERS 2022 Checklist**

|  | **Item** | **Guidance for Reporting** | **Reported in section** |
| --- | --- | --- | --- |
| **TITLE** | | |  |
| Title | 1 | Identify the study as an economic evaluation and specify the interventions being compared. | √ |
| **ABSTRACT** | | |  |
| Abstract | 2 | Provide a structured summary that highlights context, key methods, results and alternative analyses. | √ |
| **INTRODUCTION** | | | √ |
| Background and objectives | 3 | Give the context for the study, the study question and its practical relevance for decision making in policy or practice. | √ |
| **METHODS** | | |  |
| Health economic  analysis plan | 4 | Indicate whether a health economic analysis plan was developed and  where available. | √ |
| Study population | 5 | Describe characteristics of the study population (such as age range, demographics, socioeconomic, or clinical characteristics). | √ |
| Setting and location | 6 | Provide relevant contextual information that may influence findings. | √ |
| Comparators | 7 | Describe the interventions or strategies being compared and why chosen. | √ |
| Perspective | 8 | State the perspective(s) adopted by the study and why chosen. | √ |
| Time horizon | 9 | State the time horizon for the study and why appropriate. | √ |
| Discount rate | 10 | Report the discount rate(s) and reason chosen. | √ |
| Selection of outcomes | 11 | Describe what outcomes were used as the measure(s) of benefit(s) and harm(s). | √ |
| Measurement of outcomes | 12 | Describe how outcomes used to capture benefit(s) and harm(s) were measured. | √ |
| Valuation of outcomes | 13 | Describe the population and methods used to measure and value outcomes. | √ |
| Measurement and valuation of resources  and costs | 14 | Describe how costs were valued. | √ |
| Currency, price date, and conversion | 15 | Report the dates of the estimated resource quantities and unit costs, plus the currency and year of conversion. | √ |
| Rationale and  description of model | 16 | If modelling is used, describe in detail and why used. Report if the model  is publicly available and where it can be accessed. | √ |
| Analytics and assumptions | 17 | Describe any methods for analysing or statistically transforming data, any extrapolation methods, and approaches for validating any model used. | √ |
| Characterizing heterogeneity | 18 | Describe any methods used for estimating how the results of the study vary for sub-groups. | √ |
| Characterizing  distributional effects | 19 | Describe how impacts are distributed across different individuals  or adjustments made to reflect priority populations. | √ |
| Characterizing uncertainty | 20 | Describe methods to characterize any sources of uncertainty in the analysis. | √ |
| Approach to engagement with patients and others affected by the study | 21 | Describe any approaches to engage patients or service recipients, the general public, communities, or stakeholders (e.g., clinicians or payers) in the design of the study. | √ |
| **RESULTS** | | |  |
| Study parameters | 22 | Report all analytic inputs (e.g., values, ranges, references) including uncertainty or distributional assumptions. | √ |
| Summary of main results | 23 | Report the mean values for the main categories of costs and outcomes of interest and summarise them in the most appropriate overall measure. | √ |
| Effect of uncertainty | 24 | Describe how uncertainty about analytic judgments, inputs, or projections  affect findings. Report the effect of choice of discount rate and time horizon, if applicable. | √ |
| Effect of engagement with patients and others affected by the study | 25 | Report on any difference patient/service recipient, general public, community, or stakeholder involvement made to the approach or findings of the study | √ |
| **DISCUSSION** | | |  |
| Study findings, limitations, generalizability, and current knowledge | 26 | Report key findings, limitations, ethical or equity considerations not captured, and how these could impact patients, policy, or practice. | √ |
| **OTHER RELEVANT INFORMATION** | | | |
| Source of funding | 27 | Describe how the study was funded and any role of the funder in the identification, design, conduct, and reporting of the analysis | √ |
| Conflicts of interest | 28 | Report authors conflicts of interest according to journal or  International Committee of Medical Journal Editors requirements. | √ |

**Supplementary Table 2. Comparison of summary measures between original publication and reconstructed individual data**

| **Measures** | **Sintilimab plus  chemotherapy arm** | | **Placebo plus  chemotherapy arm** | |
| --- | --- | --- | --- | --- |
|  | **Original publication** | **Reconstructed data** | **Original publication** | **Reconstructed data** |
| **Overall survival** |  |  |  |  |
| Median duration (month) | 16.7 | 16.7 | 12.5 | 12.6 |
| Survival rate (12 months) | 64%^a^ | 63.90% | 52%^a^ | 52.00% |
| Survival rate (24 months) | 39%^a^ | 39.20% | 16%^a^ | 15.60% |
| **Progression free survival** |  |  |  |  |
| Median duration (month) | 7.2 | 7.2 | 5.7 | 5.8 |
| Survival rate (6 months) | 63%^a^ | 62.2% | 49%^a^ | 48.3% |
| Survival rate (12 months) | 38%^a^ | 37.6% | 15%^a^ | 15.2% |

a: Original publication was only accurate “survival rate” measures to integer digits.

The result of summary measures regarding median duration and survival rate also supported the accuracy of reconstructed KM curves (Supplementary Table 2). For example, the median duration for overall survival were identical 16.7 months in both original publication and reconstructed data in Sintilimab plus chemotherapy arm. Therefore, the reconstructed KM curves were accurate in this economic analysis, and it further ensured the reliability of results for health benefits.

**Supplementary Table 3. Estimated Parameters and Goodness of Fit from Each Survival Model.**

| **Strategies** | **Distribution** | **Parameters** | **PFS** | | | | | | **OS** | | | | | |
| --- | --- | --- | --- | --- | --- | --- | --- | --- | --- | --- | --- | --- | --- | --- |
|  |  |  | est | L95% | U95% | se | AIC | BIC | est | L95% | U95% | se | AIC | BIC |
| **Sintilimab plus chemotherapy** | **Standard parametrical models** | |  |  |  |  |  |  |  |  |  |  |  |  |
|  | **Exponential** | rate | 0.07 | 0.06 | 0.09 | 0.01 | 1392.67 | 1396.46 | 0.04 | 0.03 | 0.04 | 0.00 | 1271.28 | 1275.07 |
|  | **Gamma** | shape | 1.78 | 1.49 | 2.12 | 0.16 | 1358.55 | 1366.13 | 1.88 | 1.53 | 2.29 | 0.19 | 1240.20 | 1247.78 |
|  |  | rate | 0.15 | 0.12 | 0.19 | 0.02 |  |  | 0.09 | 0.07 | 0.12 | 0.01 |  |  |
|  | **Gen. Gamma** | mu | 2.03 | 1.82 | 2.25 | 0.11 | 1335.87 | 1347.24 | 2.99 | 2.82 | 3.15 | 0.08 | 1241.04 | 1252.41 |
|  |  | sigma | 0.94 | 0.84 | 1.05 | 0.05 |  |  | 0.83 | 0.65 | 1.05 | 0.10 |  |  |
|  |  | Q | -0.42 | -0.89 | 0.05 | 0.24 |  |  | 0.46 | -0.02 | 0.94 | 0.24 |  |  |
|  | **Gompertz** | shape | 0.03 | 0.00 | 0.05 | 0.01 | 1390.99 | 1398.57 | 0.05 | 0.03 | 0.08 | 0.01 | 1258.76 | 1266.34 |
|  |  | rate | 0.06 | 0.05 | 0.08 | 0.01 |  |  | 0.02 | 0.02 | 0.03 | 0.00 |  |  |
|  | **Weibull** | shape | 1.37 | 1.22 | 1.53 | 0.08 | 1368.74 | 1376.32 | 1.53 | 1.33 | 1.76 | 0.11 | 1243.19 | 1250.77 |
|  |  | scale | 12.68 | 11.43 | 14.06 | 0.67 |  |  | 22.11 | 19.66 | 24.87 | 1.32 |  |  |
|  | **log-Logistic** | shape | 1.91 | 1.70 | 2.14 | 0.11 | 1341.45 | 1349.03 | 1.86 | 1.62 | 2.14 | 0.13 | 1243.19 | 1244.47 |
|  |  | scale | 8.76 | 7.85 | 9.78 | 0.49 |  |  | 17.14 | 15.17 | 19.36 | 1.07 |  |  |
|  | **log-Normal** | meanlog | 2.19 | 2.08 | 2.30 | 0.06 | 1336.96 | 1344.54 | 2.88 | 2.74 | 3.02 | 0.07 | 1242.59 | 1250.17 |
|  |  | sdlog | 0.90 | 0.81 | 1.00 | 0.05 |  |  | 0.99 | 0.88 | 1.13 | 0.06 |  |  |
|  | **Royston-Parmar（1 knot）** | gamma0 | -5.39 | -6.25 | -4.52 | 0.44 | 1330.88 | 1342.25 | -5.64 | -6.86 | -4.43 | 0.62 | 1241.68 | 1253.05 |
|  |  | gamma1 | 3.17 | 2.50 | 3.84 | 0.34 |  |  | 2.34 | 1.40 | 3.29 | 0.48 |  |  |
|  |  | gamma2 | 0.30 | 0.20 | 0.40 | 0.05 |  |  | 0.13 | -0.01 | 0.27 | 0.07 |  |  |
|  | **Royston-Parmar（2 knot）** | gamma0 | -4.84 | -5.82 | -3.87 | 0.50 | **1329.90** | 1345.06 | -5.08 | -6.16 | -3.99 | 0.55 | **1234.57** | 1251.72 |
|  |  | gamma1 | 2.42 | 1.41 | 3.43 | 0.51 |  |  | 1.34 | 0.33 | 2.34 | 0.51 |  |  |
|  |  | gamma2 | -0.44 | -1.08 | 0.21 | 0.33 |  |  | -1.26 | -2.20 | -0.32 | 0.48 |  |  |
|  |  | gamma3 | 0.74 | 0.10 | 1.37 | 0.32 |  |  | 1.72 | 0.52 | 2.91 | 0.61 |  |  |
|  | **Mixture cure models** |  |  |  |  |  |  |  |  |  |  |  |  |  |
|  | **Exponential** | theta | 0.00 | 0.00 | 1.00 | NA | 1394.68 | 1402.26 | 0.00 | 0.00 | 1.00 | NA | 1273.28 | 1280.86 |
|  |  | rate | 0.07 | 0.06 | 0.09 | 0.01 |  |  | 0.04 | 0.03 | 0.04 | 0.00 |  |  |
|  | **Gamma** | theta | 0.21 | 0.15 | 0.29 | NA | 1338.22 | 1349.59 | 0.29 | 0.16 | 0.47 | NA | 1237.43 | 1248.80 |
|  |  | shape | 2.57 | 2.08 | 3.17 | 0.28 |  |  | 2.40 | 1.82 | 3.17 | 0.34 |  |  |
|  |  | rate | 0.33 | 0.24 | 0.44 | 0.05 |  |  | 0.17 | 0.10 | 0.28 | 0.04 |  |  |
|  | **Gen. Gamma** | theta | 0.16 | 0.07 | 0.33 | NA | 1335.38 | 1350.54 | 0.38 | 0.29 | 0.49 | NA | 1236.71 | 1251.87 |
|  |  | mu | 1.98 | 1.80 | 2.15 | 0.09 |  |  | 2.60 | 2.45 | 2.76 | 0.08 |  |  |
|  |  | sigma | 0.75 | 0.59 | 0.95 | 0.09 |  |  | 0.47 | 0.33 | 0.66 | 0.08 |  |  |
|  |  | Q | 0.07 | -0.51 | 0.65 | 0.30 |  |  | 1.18 | 0.52 | 1.84 | 0.34 |  |  |
|  | **Gompertz** | theta | 0.21 | 0.14 | 0.29 | NA | 1379.33 | 1390.70 | 0.40 | 0.32 | 0.48 | NA | 1241.47 | 1252.84 |
|  |  | shape | 0.11 | 0.07 | 0.14 | 0.02 |  |  | 0.15 | 0.11 | 0.19 | 0.02 |  |  |
|  |  | rate | 0.06 | 0.05 | 0.08 | 0.01 |  |  | 0.02 | 0.02 | 0.03 | 0.00 |  |  |
|  | **Weibull** | theta | 0.22 | 0.16 | 0.30 | NA | 1347.82 | 1359.19 | 0.37 | 0.27 | 0.48 | NA | 1235.02 | 1246.39 |
|  |  | shape | 1.72 | 1.52 | 1.94 | 0.11 |  |  | 1.95 | 1.65 | 2.30 | 0.17 |  |  |
|  |  | scale | 8.74 | 7.79 | 9.80 | 0.51 |  |  | 13.32 | 11.30 | 15.71 | 1.12 |  |  |
|  | **log-Logistic** | theta | 0.18 | 0.11 | 0.28 | NA | 1332.62 | 1343.99 | 0.20 | 0.07 | 0.47 | NA | 1236.83 | 1248.20 |
|  |  | shape | 2.37 | 2.03 | 2.77 | 0.19 |  |  | 2.11 | 1.72 | 2.61 | 0.23 |  |  |
|  |  | scale | 6.89 | 6.02 | 7.88 | 0.47 |  |  | 13.48 | 10.24 | 17.75 | 1.89 |  |  |
|  | **log-Normal** | theta | 0.15 | 0.08 | 0.28 | NA | 1333.44 | 1344.81 | 0.00 | 0.00 | 1.00 | NA | 1244.59 | 1255.96 |
|  |  | meanlog | 1.97 | 1.80 | 2.14 | 0.09 |  |  | 2.88 | 2.74 | 3.02 | 0.07 |  |  |
|  |  | sdlog | 0.76 | 0.66 | 0.89 | 0.06 |  |  | 0.99 | 0.88 | 1.13 | 0.06 |  |  |
|  | **Non-mixture cure models** | |  |  |  |  |  |  |  |  |  |  |  |  |
|  | **Exponential** | theta | 0.00 | 0.00 | 1.00 | NA | 1395.11 | 1402.69 | 0.00 | 0.00 | 1.00 | NA | 1273.96 | 1281.54 |
|  |  | rate | 0.00 | 0.00 | 0.01 | 0.00 |  |  | 0.00 | 0.00 | 0.01 | 0.00 |  |  |
|  | **Gamma** | theta | 0.20 | 0.14 | 0.29 | NA | 1334.39 | 1345.76 | 0.24 | 0.09 | 0.49 | NA | 1237.58 | 1248.95 |
|  |  | shape | 2.66 | 2.15 | 3.29 | 0.29 |  |  | 2.30 | 1.72 | 3.08 | 0.34 |  |  |
|  |  | rate | 0.25 | 0.17 | 0.37 | 0.05 |  |  | 0.11 | 0.05 | 0.24 | 0.04 |  |  |
|  | **Gen. Gamma** | theta | 0.15 | 0.05 | 0.36 | NA | 1334.23 | 1349.39 | 0.39 | 0.29 | 0.49 | NA | 1236.73 | 1251.89 |
|  |  | mu | 2.45 | 2.03 | 2.88 | 0.22 |  |  | 2.76 | 2.58 | 2.94 | 0.09 |  |  |
|  |  | sigma | 0.79 | 0.49 | 1.29 | 0.20 |  |  | 0.39 | 0.25 | 0.64 | 0.10 |  |  |
|  |  | Q | 0.17 | -0.58 | 0.92 | 0.38 |  |  | 1.37 | 0.50 | 2.23 | 0.44 |  |  |
|  | **Gompertz** | theta | 0.20 | 0.14 | 0.28 | NA | 1376.08 | 1387.45 | 0.40 | 0.32 | 0.48 | NA | 1240.29 | 1251.66 |
|  |  | shape | 0.15 | 0.11 | 0.19 | 0.02 |  |  | 0.17 | 0.13 | 0.21 | 0.02 |  |  |
|  |  | rate | 0.03 | 0.02 | 0.04 | 0.00 |  |  | 0.01 | 0.01 | 0.02 | 0.00 |  |  |
|  | **Weibull** | theta | 0.21 | 0.15 | 0.29 | NA | 1340.96 | 1352.33 | 0.35 | 0.23 | 0.49 | NA | 1235.51 | 1246.88 |
|  |  | shape | 1.92 | 1.70 | 2.17 | 0.12 |  |  | 2.04 | 1.71 | 2.43 | 0.18 |  |  |
|  |  | scale | 11.35 | 9.55 | 13.49 | 1.00 |  |  | 16.30 | 12.31 | 21.59 | 2.34 |  |  |
|  | **log-Logistic** | theta | 0.19 | 0.12 | 0.28 | NA | 1333.21 | 1344.58 | 0.22 | 0.09 | 0.44 | NA | 1236.52 | 1247.89 |
|  |  | shape | 2.31 | 1.97 | 2.71 | 0.19 |  |  | 2.08 | 1.68 | 2.58 | 0.23 |  |  |
|  |  | scale | 9.62 | 7.72 | 11.99 | 1.08 |  |  | 18.82 | 11.88 | 29.81 | 4.42 |  |  |
|  | **log-Normal** | theta | 0.12 | 0.05 | 0.27 | NA | 1332.39 | 1343.76 | 0.00 | 0.00 | 1.00 | NA | 1241.15 | 1252.52 |
|  |  | meanlog | 2.52 | 2.10 | 2.94 | 0.21 |  |  | 5.10 | 1.47 | 8.72 | 1.85 |  |  |
|  |  | sdlog | 0.88 | 0.72 | 1.08 | 0.09 |  |  | 1.49 | 0.87 | 2.56 | 0.41 |  |  |
| **Chemotherapy alone** | **Standard parametrical model** | |  |  |  |  |  |  |  |  |  |  |  |  |
|  | **Exponential** | rate | 0.13 | 0.11 | 0.14 | 0.01 | 1516.58 | 1520.39 | 0.06 | 0.05 | 0.06 | 0.00 | 1560.22 | 1564.03 |
|  | **Gamma** | shape | 2.01 | 1.71 | 2.35 | 0.16 | 1455.44 | 1463.05 | 1.87 | 1.57 | 2.23 | 0.17 | **1519.60** | 1527.21 |
|  |  | rate | 0.28 | 0.23 | 0.34 | 0.03 |  |  | 0.12 | 0.10 | 0.15 | 0.01 |  |  |
|  | **Gen. Gamma** | mu | 1.83 | 1.67 | 1.98 | 0.08 | 1450.87 | 1462.29 | 2.70 | 2.54 | 2.85 | 0.08 | 1521.37 | 1532.78 |
|  |  | sigma | 0.78 | 0.70 | 0.87 | 0.04 |  |  | 0.76 | 0.63 | 0.93 | 0.08 |  |  |
|  |  | Q | 0.27 | -0.07 | 0.61 | 0.17 |  |  | 0.62 | 0.19 | 1.06 | 0.22 |  |  |
|  | **Gompertz** | shape | 0.06 | 0.03 | 0.09 | 0.01 | 1501.61 | 1509.22 | 0.06 | 0.04 | 0.09 | 0.01 | 1537.02 | 1544.63 |
|  |  | rate | 0.09 | 0.08 | 0.11 | 0.01 |  |  | 0.03 | 0.03 | 0.04 | 0.00 |  |  |
|  | **Weibull (AFT)** | shape | 1.47 | 1.34 | 1.62 | 0.07 | 1466.44 | 1474.05 | 1.51 | 1.35 | 1.70 | 0.09 | 1521.86 | 1529.47 |
|  |  | scale | 7.99 | 7.34 | 8.70 | 0.35 |  |  | 16.38 | 14.93 | 17.98 | 0.78 |  |  |
|  | **Weibull (PH)** | shape | 1.47 | 1.34 | 1.62 | 0.07 | 1466.44 | 1474.05 | 1.51 | 1.35 | 1.70 | 0.09 | 1521.86 | 1529.47 |
|  |  | scale | 0.05 | 0.03 | 0.07 | 0.01 |  |  | 0.01 | 0.01 | 0.02 | 0.00 |  |  |
|  | **log-Logistic** | shape | 2.16 | 1.95 | 2.39 | 0.11 | **1444.32** | 1455.94 | 1.92 | 1.70 | 2.15 | 0.11 | 1520.26 | 1527.88 |
|  |  | scale | 5.73 | 5.23 | 6.28 | 0.27 |  |  | 12.35 | 11.12 | 13.71 | 0.66 |  |  |
|  | **log-Normal** | meanlog | 1.73 | 1.63 | 1.82 | 0.05 | 1451.28 | 1458.89 | 2.51 | 2.40 | 2.63 | 0.06 | 1527.62 | 1535.23 |
|  |  | sdlog | 0.82 | 0.75 | 0.89 | 0.04 |  |  | 0.94 | 0.85 | 1.04 | 0.05 |  |  |
|  | **Royston-Parmar（1 knot）** | gamma0 | -3.71 | -4.24 | -3.19 | 0.27 | 1453.48 | 1464.89 | -4.75 | -5.59 | -3.90 | 0.43 | 1521.21 | 1532.62 |
|  |  | gamma1 | 2.46 | 1.88 | 3.03 | 0.29 |  |  | 2.03 | 1.35 | 2.71 | 0.35 |  |  |
|  |  | gamma2 | 0.14 | 0.07 | 0.22 | 0.04 |  |  | 0.07 | -0.02 | 0.16 | 0.05 |  |  |
|  | **Royston-Parmar（2 knot）** | gamma0 | -3.65 | -4.18 | -3.13 | 0.27 | 1453.96 | 1469.18 | -4.63 | -5.51 | -3.76 | 0.44 | 1522.83 | 1538.05 |
|  |  | gamma1 | 2.14 | 1.35 | 2.93 | 0.40 |  |  | 1.82 | 0.91 | 2.72 | 0.46 |  |  |
|  |  | gamma2 | -0.21 | -0.68 | 0.26 | 0.24 |  |  | -0.15 | -0.67 | 0.38 | 0.27 |  |  |
|  |  | gamma3 | 0.42 | -0.15 | 0.99 | 0.29 |  |  | 0.27 | -0.40 | 0.94 | 0.34 |  |  |
|  | **Mixture cure models** | |  |  |  |  |  |  |  |  |  |  |  |  |
|  | **Exponential** | theta | 0.00 | 0.00 | 1.00 | NA | 1518.59 | 1526.20 | 0.00 | 0.00 | 1.00 | NA | 1562.23 | 1569.84 |
|  |  | rate | 0.13 | 0.11 | 0.14 | 0.01 |  |  | 0.06 | 0.05 | 0.06 | 0.00 |  |  |
|  | **Gamma** | theta | 0.06 | 0.03 | 0.12 | NA | 1444.45 | 1455.86 | 0.03 | 0.00 | 0.90 | NA | 1521.49 | 1532.90 |
|  |  | shape | 2.36 | 1.99 | 2.81 | 0.21 |  |  | 1.92 | 1.52 | 2.43 | 0.23 |  |  |
|  |  | rate | 0.37 | 0.30 | 0.46 | 0.04 |  |  | 0.13 | 0.08 | 0.21 | 0.03 |  |  |
|  | **Gen. Gamma** | theta | 0.06 | 0.03 | 0.12 | NA | 1446.34 | 1461.56 | 0.01 | 0.00 | 1.00 | NA | 1523.38 | 1538.60 |
|  |  | mu | 1.87 | 1.73 | 2.01 | 0.07 |  |  | 2.69 | 2.48 | 2.90 | 0.11 |  |  |
|  |  | sigma | 0.63 | 0.53 | 0.75 | 0.06 |  |  | 0.76 | 0.58 | 1.00 | 0.11 |  |  |
|  |  | Q | 0.72 | 0.29 | 1.15 | 0.22 |  |  | 0.63 | 0.13 | 1.13 | 0.26 |  |  |
|  | **Gompertz** | theta | 0.07 | 0.04 | 0.12 | NA | 1469.75 | 1481.16 | 0.16 | 0.08 | 0.29 | NA | 1535.28 | 1546.69 |
|  |  | shape | 0.16 | 0.12 | 0.19 | 0.02 |  |  | 0.10 | 0.06 | 0.13 | 0.02 |  |  |
|  |  | rate | 0.07 | 0.06 | 0.09 | 0.01 |  |  | 0.03 | 0.03 | 0.05 | 0.00 |  |  |
|  | **Weibull** | theta | 0.07 | 0.04 | 0.12 | NA | 1445.93 | 1457.34 | 0.10 | 0.02 | 0.37 | NA | 1522.71 | 1534.12 |
|  |  | shape | 1.72 | 1.55 | 1.91 | 0.09 |  |  | 1.60 | 1.38 | 1.86 | 0.12 |  |  |
|  |  | scale | 6.97 | 6.41 | 7.58 | 0.30 |  |  | 14.38 | 11.62 | 17.80 | 1.56 |  |  |
|  | **log-Logistic** | theta | 0.00 | 0.00 | 1.00 | NA | 1450.31 | 1461.73 | 0.00 | 0.00 | 1.00 | NA | 1522.29 | 1533.71 |
|  |  | shape | 2.17 | 1.92 | 2.45 | 0.13 |  |  | 1.92 | 1.70 | 2.16 | 0.12 |  |  |
|  |  | scale | 5.71 | 5.13 | 6.35 | 0.31 |  |  | 12.33 | 11.07 | 13.73 | 0.68 |  |  |
|  | **log-Normal** | theta | 0.01 | 0.00 | 0.56 | NA | 1453.21 | 1464.62 | 0.00 | 0.00 | 1.00 | NA | 1529.64 | 1541.05 |
|  |  | meanlog | 1.71 | 1.59 | 1.83 | 0.06 |  |  | 2.51 | 2.40 | 2.63 | 0.06 |  |  |
|  |  | sdlog | 0.80 | 0.72 | 0.90 | 0.05 |  |  | 0.94 | 0.85 | 1.04 | 0.05 |  |  |
|  | **Non-mixture cure models** | |  |  |  |  |  |  |  |  |  |  |  |  |
|  | **Exponential** | theta | 0.00 | 0.00 | 1.00 | NA | 1519.26 | 1526.87 | 0.00 | 0.00 | 1.00 | NA | 1562.78 | 1570.39 |
|  |  | rate | 0.00 | 0.00 | 0.01 | 0.00 |  |  | 0.00 | 0.00 | 0.01 | 0.00 |  |  |
|  | **Gamma** | theta | 0.03 | 0.01 | 0.11 | NA | 1446.97 | 1458.39 | 0.01 | 0.00 | 0.82 | NA | 1521.54 | 1532.95 |
|  |  | shape | 2.22 | 1.85 | 2.67 | 0.21 |  |  | 1.82 | 1.41 | 2.33 | 0.23 |  |  |
|  |  | rate | 0.17 | 0.11 | 0.28 | 0.04 |  |  | 0.05 | 0.01 | 0.19 | 0.03 |  |  |
|  | **Gen. Gamma** | theta | 0.07 | 0.03 | 0.13 | NA | 1447.44 | 1462.66 | 0.00 | 0.00 | 1.00 | NA | 1523.39 | 1538.61 |
|  |  | mu | 2.42 | 2.25 | 2.59 | 0.09 |  |  | 4.31 | -1.50 | 10.12 | 2.96 |  |  |
|  |  | sigma | 0.46 | 0.29 | 0.73 | 0.11 |  |  | 0.99 | 0.16 | 6.26 | 0.93 |  |  |
|  |  | Q | 1.22 | 0.45 | 2.00 | 0.40 |  |  | 0.48 | -1.02 | 1.98 | 0.77 |  |  |
|  | **Gompertz** | theta | 0.07 | 0.04 | 0.11 | NA | 1464.24 | 1475.65 | 0.15 | 0.07 | 0.29 | NA | 1533.68 | 1545.09 |
|  |  | shape | 0.22 | 0.18 | 0.26 | 0.02 |  |  | 0.12 | 0.09 | 0.16 | 0.02 |  |  |
|  |  | rate | 0.02 | 0.02 | 0.03 | 0.00 |  |  | 0.01 | 0.01 | 0.02 | 0.00 |  |  |
|  | **Weibull** | theta | 0.06 | 0.03 | 0.12 | NA | 1445.75 | 1457.16 | 0.05 | 0.00 | 0.56 | NA | 1521.96 | 1533.37 |
|  |  | shape | 1.91 | 1.70 | 2.14 | 0.11 |  |  | 1.69 | 1.43 | 1.99 | 0.14 |  |  |
|  |  | scale | 11.38 | 9.16 | 14.14 | 1.26 |  |  | 27.18 | 12.10 | 61.06 | 11.22 |  |  |
|  | **log-Logistic** | theta | 0.02 | 0.00 | 0.09 | NA | 1447.48 | 1458.90 | 0.01 | 0.00 | 0.74 | NA | 1521.75 | 1533.17 |
|  |  | shape | 1.97 | 1.72 | 2.26 | 0.14 |  |  | 1.71 | 1.41 | 2.08 | 0.17 |  |  |
|  |  | scale | 12.70 | 9.03 | 17.86 | 2.21 |  |  | 34.31 | 12.29 | 95.78 | 17.97 |  |  |
|  | **log-Normal** | theta | 0.00 | 0.00 | 0.61 | NA | 1449.38 | 1460.79 | 0.00 | 0.87 | 0.00 | NA | 1521.47 | 1532.89 |
|  |  | meanlog | 3.49 | 2.31 | 4.68 | 0.60 |  |  | 7.63 | -0.63 | 15.89 | 4.22 |  |  |
|  |  | sdlog | 1.24 | 0.95 | 1.62 | 0.17 |  |  | 2.01 | 1.01 | 3.98 | 0.70 |  |  |

**Supplementary Table 4. Probability and Costs Related to Subsequent Therapy.**

| **Parameter** | **Base case (Range)** | **Distribution** | **Source** |
| --- | --- | --- | --- |
| **Probabilities in sintilimab plus chemotherapy strategy (%)** |  |  |  |
| Anti-PD-(L)1 agent | 13.0 (9.8 to 16.3) | Beta | 1 |
| Targeted drug therapy | 8.0 (6.0 to 10.0) | Beta | 1 |
| Chemotherapy | 20.0 (15.0 to 25.0) | Beta | 1 |
| **Probabilities in chemotherapy strategy (%)** |  |  |  |
| Anti-PD-(L)1 agent | 18.0 (13.5 to 22.5) | Beta | 1 |
| Targeted drug therapy | 12.0 (15.0 to 10.9) | Beta | 1 |
| Chemotherapy | 23.0 (17.3 to 28.8) | Beta | 1 |
| **Costs per event, $** |  |  |  |
| Anti-PD-(L)1 agent (camrelizumab per 100 mg) ^a, 3^ | 226.98 (170.24 to 283.73) | Gamma | 2 |
| Targeted drug therapy (anlotinib per 10 mg) ^a, 3^ | 39.53 (29.65 to 49.41) | Gamma | 2 |
| Chemotherapy (docetaxel per 100 mg) ^a, 3^ | 230.36 (172.77 to 287.95) | Gamma | 2 |

^a^ According to the recommendation of Chinese Society of Clinical Oncology Guidelines (2022), patients with OSCC who have progressed can be treated with camrelizumab as an anti-PD-(L)1 agent, anlotinib as a targeted drug therapy, and docetaxel as a chemotherapy.

1. Lu Z, Wang J, Shu Y, et al. Sintilimab versus placebo in combination with chemotherapy as first line treatment for locally advanced or metastatic oesophageal squamous cell carcinoma (ORIENT-15): multicentre, randomised, double blind, phase 3 trial. *BMJ (Clinical research ed)*. Apr 19 2022;377:e068714. doi:10.1136/bmj-2021-068714
2. Chinese drug (2021). Chinese Drug Price of Drug Centralized Bid Procurement. Available at: <https://db.yaozh.com/yaopinzhongbiao> (accessed Sep 30, 2022).
3. Chinese Society of Clinical Oncology (2022). CSCO Guidelines in Diagnosis and Treatment of Esophageal Cancer. http://www.csco.org.cn/cn/index.aspx (Accessed on April 11, 2022).

**Supplementary Table 5. Probability and Costs Related to Adverse Events**

| **Parameter** | **Base case (Range)** | **Distribution** | **Source** |
| --- | --- | --- | --- |
| **Probabilities in sintilimab plus chemotherapy strategy (%)** |  |  |  |
| Anaemia | 14.0 (10.5 to 17.5) | Beta | 1 |
| White blood cell count decreased | 17.0 (12.8 to 21.3) | Beta | 1 |
| Neutrophil count decreased | 30.0 (22.5 to 37.5) | Beta | 1 |
| Hyponatraemia | 5.0 (3.8 to 6.3) | Beta | 1 |
| Hypokalaemia | 8.0 (6.0 to 10.0) | Beta | 1 |
| Lymphocyte count decreased | 5.0 (3.8 to 6.3) | Beta | 1 |
| **Probabilities in chemotherapy strategy (%)** |  |  |  |
| Anaemia | 13.0 (9.8 to 16.3) | Beta | 1 |
| White blood cell count decreased | 22.0 (16.5 to 27.5) | Beta | 1 |
| Neutrophil count decreased | 34.0 (25.5 to 42.5) | Beta | 1 |
| Hyponatraemia | 3.0 (2.3 to 3.8) | Beta | 1 |
| Hypokalaemia | 4.0 (3.0 to 5.0) | Beta | 1 |
| Lymphocyte count decreased | 5.0 (3.8 to 6.3) | Beta | 1 |
| **Costs per event, $** |  |  |  |
| Anaemia | 531.7 (398.8 to 664.6) | Gamma | 2 |
| White blood cell count decreased | 466.0 (349.5 to 582.5) | Gamma | 3 |
| Neutrophil count decreased | 461.5 (346.1 to 576.9) | Gamma | 2 |
| Hyponatraemia | 23.3 (17.4 to 29.1) | Gamma | 4 |
| Hypokalaemia | 15.8 (11.8 to 19.7) | Gamma | 4 |
| Lymphocyte count decreased | 529.8 (397.4 to 662.3) | Gamma | 4 |

1. Lu Z, Wang J, Shu Y, et al. Sintilimab versus placebo in combination with chemotherapy as first line treatment for locally advanced or metastatic oesophageal squamous cell carcinoma (ORIENT-15): multicentre, randomised, double blind, phase 3 trial. *BMJ (Clinical research ed)*. Apr 19 2022;377:e068714. doi:10.1136/bmj-2021-068714
2. Wu B, Dong B, Xu Y, Zhang Q, Shen J, Chen H, Xue W. Economic evaluation of first-line treatments for metastatic renal cell carcinoma: a cost-effectiveness analysis in a health resource-limited setting. PloS one 2012, 7(3): e32530.
3. Bai Y, Xu Y, Wu B. Cost–effectiveness and budget impact analysis of apatinib for advanced metastatic gastric cancer from the perspective of health insurance system. Gastroent. Res. Pract. 2017(1), 1–7 (2017).
4. Drug and medical service price. https://www.yaozh.com/. Accessed Oct 5. 2022
